# Calcitonin concentrations in patients with chronic kidney disease on hemodialysis in reference to parathyroidectomy

**DOI:** 10.1186/s13104-019-4479-6

**Published:** 2019-07-19

**Authors:** Roza Sabia, Martin Wagner, Kristina Susa, Johannes Lemke, Lars Rothermund, Doris Henne-Bruns, Andreas Hillenbrand

**Affiliations:** 1 Department of Internal Medicine I, University Hospital Ulm, Albert-Einstein-Allee 23, 89081 Ulm, Germany; 2 Department of General and Visceral Surgery, University Hospital Ulm, Albert-Einstein-Allee 23, 89081 Ulm, Germany; 3 KfH Kuratorium für Dialyse und Nierentransplantation e.V, KfH-Nierenzentrum, Erlenstraße 40, 89077 Ulm, Germany

**Keywords:** Calcitonin, Hypercalcitoninemia, Secondary hyperparathyroidism, Renal insufficiency, Parathyroid surgery

## Abstract

**Objective:**

Calcitonin is considered to be a biomarker of medullary thyroid carcinoma and C-cell hyperplasia, but calcitonin can also be elevated in about 30% of the patients with end-stage kidney disease. We reported preoperative calcitonin serum levels in 31 patients on hemodialysis before parathyroid surgery, evaluate influencing factors on calcitonin levels and determine postoperative calcitonin levels after parathyroid surgery.

**Results:**

Median preoperative serum calcitonin was 8 pg/ml (range 2 to 165 pg/ml), serum calcitonin concentration declined postoperatively to 3 pg/ml (range 1 to 192 pg/ml). There was a strong positive correlation between pre- and postoperative serum calcitonin levels (r = 0.92; p > 0.01). Elevated preoperative serum calcitonin concentrations (more than 10 pg/ml) were found in 14 out of 32 dialysis-dependent patients (44%). Preoperative calcitonin levels of male patients were significantly higher than female patients. Hypercalcitoninemia showed a positive correlation to body mass index (p < 0.01). We found no correlation between preoperative calcitonin levels and patients’ age, duration of hemodialysis dependency, cinacalcet medication or preoperative concentrations of parathyroid hormone, creatinine and calcium. Basal calcitonin levels higher than 10 pg/ml are common in patients on dialysis. Male gender and morbid obesity are risk factors for hypercalcitoninemia. Calcitonin concentration decreases after parathyroid operation.

## Introduction

Calcitonin is a 32 amino acid polypeptide hormone secreted in humans primarily by the thyroid C-cells. It protects against the development of hypercalcemia. When levels of calcium in the blood increase, calcitonin is secreted in higher quantities. Further, it is considered to be a biomarker of C-cell disease (medullary thyroid carcinoma (MTC) and C-cell hyperplasia (CCH)) [1]. Calcitonin measurement is strongly recommended for both initial diagnosis and monitoring of patients with residual and recurrent MTC [2]. Assaying calcitonin before routine thyroid or parathyroid surgery may help with early identification of MTC [3]. The normal range of basal calcitonin (bCT) is < 10 pg/ml. It is known that men have twice as many C-cells as women. Gender-specific bCT cut-offs are defined and should be used in the clinical routine. Elevated serum calcitonin values of >  30 pg/ml in female and >  60 pg/ml in male patients make sporadic medullary thyroid cancer (MTC) suspect. In order to obtain better survival of early micrometastasis due to MTC, curative surgery is recommended [1, 4, 5]. Increased calcitonin levels can be observed in other circumstances than MTC and CCH, for instance other endocrine tumors, autoimmune thyroid disease, hypergastrinemia or sepsis [6]. Elevated calcitonin levels have also been reported in patients with end-stage chronic kidney disease (CKD), detected before the uremic phase and in the absence of hypercalcemia. Depending on the data source, 30–46% of CKD patients can be affected [7–9].

An uncertainty remains regarding the proper therapeutic strategy among CKD patients with hypercalcitoninemia. Preoperative serum calcitonin levels in patients with secondary hyperparathyroidism are seldom reported and parameters of influence are not investigated. Further, the postoperative course of calcitonin in CKD patients after parathyroidectomy has not been analyzed to date.

We reported preoperative calcitonin serum levels in a patient collective with CKD on hemodialysis before parathyroid surgery. Moreover, we evaluated influencing factors such as cinacalcet medication, gender, body mass index, parathyroid hormone (PTH) levels or length of dialysis. Further, we determined postoperative calcitonin levels after parathyroid surgery.

## Main text

### Patients

From years 2013 to 2018, 36 CKD patients with secondary hyperparathyroidism were referred to University Hospital Ulm for parathyroidectomy. Inclusion criteria were dialysis-dependent end-stage renal disease and parathyroid resection due to secondary hyperparathyroidism. We performed preoperative screening and postoperative follow-up including measurement of calcitonin, PTH and calcium. Five patients had to be excluded as they passed away in the meantime (3) or received a kidney transplantation (2). Therefore, we included altogether 31 patients in the analysis.

Anthropomorphic data and further patients’ details are listed in Table 1. Serum calcitonin, calcium, creatinine, PTH and patients’ medication history were included in both preoperative and postoperative evaluation. The study was performed with the permission of the independent local ethics committee of the University of Ulm (approval 405/18). An informed consent of all patients was obtained.

**Table 1:** Anthropomorphic data and further patient details of 31 patients and correlation to preoperative calcitonin level

Anthropomorphic date	Results (median/range)	Correlation with preoperative calcitonin
Gender (male/female)	20 male 11 female	*r = 0.53* *p < 0.01*
Age at operation	59 years (range 25–78)	r = 0.07 p = 0.35
Preoperative duration of dialysis	9 years (range 19 months to 22 years)	r = 0.04 p = 0.42
Body mass index	25 kg/m^2^ (range 18 to 47)	*r = 0.46* *p < 0.01*
Patients on cinacalcet medication	12 patients	r = 0.02 p = 0.46
Preoperative creatinine highest level	851 μmol/l (range 566 to 1389)	r = 0.11 p = 0.27
Preoperative calcium level	2.6 mmol/l (range 2.0 to 2.9)	r = 0.15 p = 0.21
Parathyroid hormone		
Preoperative parathyroid hormone	926 pg/ml (range 371 to 2146)^a^	r = 0.09 p = 0.38
Direct postoperative parathyroid hormone	<5 pg/ml (range < 5 to 167)	
Postoperative parathyroid hormone during course	42 pg/ml (range < 5 to 625)	
Calcitonin		
Preoperative calcitonin	8 pg/ml (range 2 to 165)	
Postoperative calcitonin during course	3 pg/ml (range 1 to 192)	

MTC, medullary thyroid carcinoma; CKD, chronic kidney disease; pg/ml, picogram per milliliter; bCT, basal calcitonin; CCH, C-cell hyperplasia; kg/m^2^, kilogram per square meter; PTH, parathyroid hormone; BMI, body mass index

^a^One patient suffered from persistent hyperparathyroidism after incomplete previous operation (PTH: 412 pg/ml)

Preoperative ultrasound examination revealed thyroid and parathyroid gland size as well as suspicious thyroid nodules. No further imaging of the parathyroid glands was routinely prompted. We gained the preoperative blood samples before a dialysis. The day before the operation the last preoperative hemodialysis took place. All surgical interventions were performed by three consultant surgeons, who are specialized in thyroid surgery with a standard collar incision. Intraoperatively, all parathyroid glands could be identified. In 10 out of 31 patients, all parathyroid glands were removed without auto-transplantation. In 21 patients, a subtotal resection was performed by leaving a small portion of a lower parathyroid gland in the neck (n = 10) or re-implantation in the forearm within the brachioradialis muscle (n = 11). PTH levels were measured intraoperatively after removal of parathyroid glands. The resected tissue was histologically verified initially by intraoperative frozen section and subsequently using conventional histological testing. An additional thyroid resection was performed in 13 patients mainly based on evidence of suspicious nodules in the preoperative ultrasound examination. A complete thyroidectomy was performed in 3 patients, a hemithyroidectomy with subtotal resection of the other side in 2 patients. Four patients received a hemithyroidectomy and in another 4 patients we performed a unilateral subtotal resection. In 2 out of 3 patients with complete thyroidectomy, a papillary carcinoma was found. No resected thyroid nodule was suspicious for MTC.

Depending on the postoperative calcium und PTH concentration, a calcitriol and calcium substitution per os was partially indicated. The therapeutic goal in these cases was reaching serum calcium levels within the normal range.

### Statistical evaluation

Calcitonin concentrations at screening and in follow-up were measured by three different assays, which corresponded to the WHO Standards. After 31.01.2017 an e801-module of the ElectroChemiLuminescence Immunoassay “ECLIA” at Roche immunoassay analyzers Cobas 8000 was used. From 2016 to 2017, e602-module was used. From 2013 to 2016 Siemens Immunoassay Analyseautomaten Immulite 1000 was used. All values are expressed as median with range.

When comparing data, statistical analysis was performed using the paired *t* test (normally distributed data) or the Wilcoxon signed rank test (non-parametric data).

The Spearman rank-order correlation coefficient was calculated for correlation analysis. R indicates the correlation coefficient. A value of p < 0.05 was considered statistically significant. Correlation coefficient values between 0.3 and 0.7 indicate a moderate relationship, values between 0.7 and 1.0 indicate a strong, positive linear relationship.

In boxplots, the top and bottom of the rectangle represent the 25th and 75th percentile; whiskers represent the 5th and the 95th percentile, respectively. The line within the rectangle represents the median.

Analysis was performed with WinSTAT, Version 2009.1 (R. Fitch Software) and the IBM SPSS Statistics 20.0.0 (Ehningen, Germany).

### Results

**Fig. 1:**
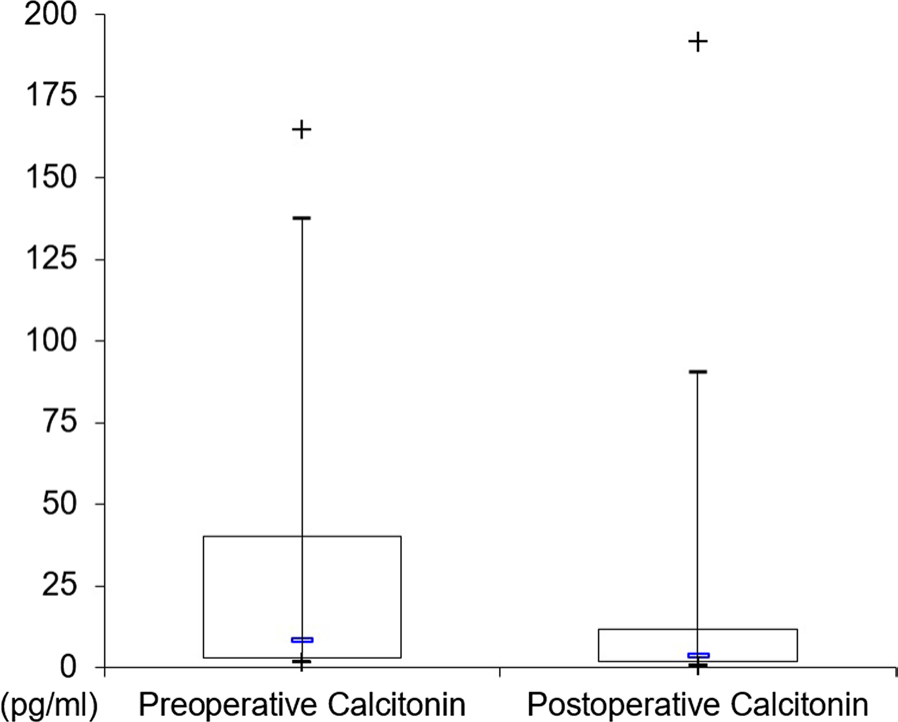
Preoperative and postoperative calcitonin levels of 31 patients on dialysis

The median preoperative serum calcitonin was 8 pg/ml (range 2 to 165 pg/ml); the serum levels declined postoperatively up to 3 pg/ml (range 1 to 192 pg/ml; Fig. 1). Elevated preoperative serum calcitonin concentrations were found in 14 out of 32 dialysis dependent patients (44%). Five male patients had preoperative calcitonin levels of 60 pg/ml and above. Preoperative calcitonin levels of male patients were significantly higher than female patients (median male serum calcitonin: 18.8 pg/ml; range 2.2 to 165 pg/ml; median female serum calcitonin: 3.2 pg/ml; range 1.6 to 51 pg/ml; p < 0.01). Further, calcitonin levels showed a strong positive correlation to body mass index (BMI; p < 0.01, Table 1). We found no correlation between preoperative calcitonin levels and patients’ age at operation, duration of hemodialysis dependency, cinacalcet medication or preoperative concentrations of PTH, creatinine and calcium.

In the postoperative course of median 33 months (range 4 to 69 months), calcitonin levels decreased in all but in one patient (Fig. 1). Median serum calcitonin levels (3 pg/ml; range 1 to 192 pg/ml) were within normal range in 24 patients. This patient sample included all female patients (n = 11). Seven male patients showed elevated postoperative levels up to 192 pg/ml. There was a strong positive correlation between pre- and postoperative serum calcitonin levels (r = 0.92; p > 0.01).

### Discussion

Patients on hemodialysis with secondary hyperparathyroidism often present with elevated levels of calcitonin. In our analysis, we found elevated calcitonin levels in almost half of patients, which is comparable with literature [8]. Five male and one female patient (16%) showed calcitonin levels above 60 pg/ml or 30 pg/ml, respectively. Such diagnostic findings indicate in general a recommendation for thyroidectomy. Those common recommendations based alone on calcitonin concentrations cannot be transferred by implication in patients on dialysis. The causes of hypercalcitoninemia in patients with CKD are not clearly understood. It is assumed that an increased C-cell hormone secretion results from disturbance in mineral metabolism due to renal failure. Elevated calcitonin serum concentrations in patients with CKD do not automatically indicate a medullary thyroid carcinoma but rather a changed calcium homeostasis [9]. Increase of serum calcium concentration stimulates the calcitonin secretion. Otherwise calcitonin protects against the development of hypercalcemia [10]. Further, a regulating role of calcitonin in vitamin D metabolism is described. Calcitonin is known to stimulate 1,25D production in the proximal straight tubule of the kidney. On the other hand, 1,25D suppresses the calcitonin secretion as a component of a feedback loop [11]. Elevated calcitonin levels in dialysis-dependent patients could be explained either by elevated C-cell production or by calcitonin retention due to a decrease in renal function (kidney as primary site for metabolism of calcitonin) [10, 12]. Preoperative elevated calcitonin levels decreased after parathyroid operation and often normalize after successful kidney transplantation [13]. Therefore, elevated calcitonin levels are presumable explained by an increased calcitonin secretion due to altered calcum/parathormone/1,25D homeostasis.

To exclude concomitant thyroid disease, we performed a preoperative ultrasound in every patient in particular since patients undergoing hemodialysis frequently develop thyroid abnormalities [14]. Preoperative ultrasound reviewed suspicious thyroid nodes especially well circumscribed nodes at the junction of the upper and middle third of the lateral lobe of the thyroid, possibly suspect for medullary thyroid cancer. In all patients with suspicious nodes found by ultrasound, a (partial) thyroid resection was performed. Two out of these 31 patients were found to suffer from papillary thyroid cancer, but in no patient was a medullary thyroid cancer found.

In order to understand why some CKD patients present with elevated serum calcitonin levels and others not, we analyzed the possible risk factors for elevated calcitonin levels. We found male gender and high BMI to be risk factors. Morbidly obese patients with BMI > 30 kg/m^2^ were especially prone to elevated calcitonin levels (n = 8; median preoperative calcitonin: 32 pg/ml; range 2 to 123 pg/ml). Contrary to our previous assumption, we found no correlation of preoperative calcitonin levels with PTH or time of dialysis.

Which calcitonin concentration is suspicious for medullary thyroid carcinoma in CKD patients? In our cohort, we found no patients suffering from MTC. All patients except one had lowered calcitonin concentration over time, making MTC unlikely. The patient with calcitonin rising from 165 to 192 pg/ml was re-evaluated in this study by ultrasound (thyroid volume 18 ml, no suspicious thyroid nodules) and by calcium stimulation test (no relevant increase to 231 pg/ml). In this patient (male; BMI: 26 kg/m^2^), the increase could be partially explained by recurrent hyperparathyroidism 2 years after initial operation (PTH postoperative: 29 pg/ml; over the time course 419 pg/ml).

Elevated calcitonin levels in hemodialysis patients are not a surrogate marker for hyperparathyroidism but for medullary thyroid cancer. But the recommended thyroidectomy threshold is higher in patents with CKD compared to patients with normal renal function. Borchart et al. reported, that medullary thyroid carcinoma could be found in CKD patients only when unstimulated calcitonin concentration exceeded 250 pg/ml. 40 patients were reported with CKD and maximum pentagastrin-stimulated calcitonin concentration greater than 100 pg/ml. A thyroidectomy was performed in 22 out of these 40 patients. Seven of them presented with a MTC, the rest showed C-cell hyperplasia. MTC was found in patients on dialysis only when unstimulated calcitonin concentration exceeded 250 pg/ml and a maximum pentagastrin-stimulated calcitonin concentration was greater than 400 pg/ml. Those values are reported to be a clinically meaningful threshold for thyroidectomy [15].

Our results lead to a similar conclusion. Namely basal calcitonin concentrations higher than 10 pg/ml are common in patients with CKD on dialysis and even unstimulated calcitonin levels greater 100 pg/ml are not necessarily associated with a MTC. Whether increased calcitonin levels have any role in chronic kidney disease remains unknown [10]. The important finding of this paper is the almost universal decrease of calcitonin following parathyroidectomy in hemodialysis patients. This allows, in unclear cases with preoperative, relevant elevated calcitonin levels, a postoperative calcitonin control. A medullary thyroid carcinoma should be taken into consideration if the calcitonin levels have not dropped significantly. Male gender and morbid obesity are risk factors for elevated calcitonin levels.

## Limitations

- Presented study is a retrospective study with inferior level of evidence compared with prospective studies.
- PTH fluctuates over a very wide range. In our analysis, we used preoperative PTH levels determined directly before dialysis (repeated measurements). The concentrations, although measured within a very narrow time frame, differed up to three-fold in the same patient. However, no correlation was also seen in the evaluation of the highest or lowest PTH value during the last month prior to surgery.
- We concentrated our investigations on the measurement of basal calcitonin. We did not perform a calcitonin stimulation test.
- An additional thyroid resection was performed in 13 patients. A complete thyroidectomy was performed in 3 patients (2× papillary thyroid carcinoma; 1× III° nontoxic nodal goiter). Pre- and postoperative calcitonin levels of these 3 patients were approximately unchanged between 2 and 4 pg/ml. Pre- and postoperative calcitonin levels of all 13 patients with thyroid resection did not differ significantly from levels of the entire group (preoperative median level 4 pg/ml; range 2 to 123 pg/ml; postoperative median level 3 pg/ml; range 1 to 36 pg/ml).

## Data Availability

The clinical data used to support the findings of this study are restricted by the Ethical committee of the University of Ulm in order to protect patient privacy. Data are available from corresponding author for researchers who meet the criteria for access to confidential data.

## Abbreviations

MTC: medullary thyroid carcinoma
CKD: chronic kidney disease
MTC: medullary thyroid carcinoma
CCH: C-cell hyperplasia
bCT: basal calcitonin
PTH: parathyroid hormone
BMI: body mass index
